# Surface Modification and Properties of Thin Ink Films with Added TiO_2_ and ZnO Nanoparticles Applied on Paperboard Substrates

**DOI:** 10.3390/ma16020478

**Published:** 2023-01-04

**Authors:** Sanja Mahović Poljaček, Tamara Tomašegović, Maja Strižić Jakovljević, Davor Donevski

**Affiliations:** Faculty of Graphic Arts, University of Zagreb, 10000 Zagreb, Croatia

**Keywords:** surface modification, ink films, TiO_2_ and ZnO nanoparticles, paperboard

## Abstract

In this study, the surface modification of thin ink films with added nanoparticles was used to improve the functional properties of ink applied on paperboard substrates. The surface modification was performed by additional exposure of the samples to xenon radiation. Anatase TiO_2_, rutile TiO_2_ and ZnO were added to the base ink. The effect of surface modification on the surface, structural, and mechanical properties of the printed ink films was determined by FTIR-ATR spectroscopy, calculating the surface free energy and adhesion parameters, performing the rub resistance test of the printed samples, and by measuring the resistance to bending. Color measurements on the ink films were performed in order to observe the optical properties of unmodified and modified samples. The results showed that surface modification significantly improved the adhesion properties of the thin ink films and the mechanical properties of the samples. The results obtained on uncoated and coated paperboard showed that the addition of rutile TiO_2_ and ZnO nanoparticles had the greatest effect on the rub resistance of the ink films. The results of the color analysis showed that the addition of nanoparticles did not change the optical properties of the modified ink films and that rutile TiO_2_ and ZnO nanoparticles improved the lightfastness of the applied ink films.

## 1. Introduction

Research in the field of the synthesis and modification of deposited films with nanoscale materials is widespread. By adding nanoscale materials to the base material, it is possible to improve the properties of films, extend their range of application, and give them new functionalities. Nowadays, modern technology makes it possible to adjust the properties of materials to be used as ink films and coatings by the addition of nanoparticles and to tailor their properties to the specific needs of different applications. By adjusting their properties, they can even be used as multipurpose films. The areas in which nanomaterials are used are numerous due to the wide range of possible applications for the deposition of various coatings and films. The automotive industry, cosmetics, machinery, furniture, structural materials, agriculture, food and food safety, pharmaceuticals, and medicine are some areas where nanomaterials are used [1,2,3]. 

In addition, nanoscale materials are found in security prints, paints, inks, and coatings used in the packaging industry, where the potential benefits are often related to the various properties of the packaging, such as corrosion protection, antibacterial, fire retardant, self-cleaning, UV protection, scratch resistance, and other protective functions [4,5,6,7]. Furthermore, the use of nanomaterials in the printing ink and packaging industries is of great interest, as they can improve the properties of applied ink films and coatings, enhance the interaction between the materials in contact, and, among other things, protect the product from different production stages and environmental conditions [3].

Currently, important nanomaterials used in the ink and paint industry are nanoscale titanium dioxide (TiO_2_), silicon dioxide (SiO_2_), zinc oxide (ZnO), aluminum oxide (Al_2_O_3_), silver oxide (Ag_2_O), and others [8]. 

It has already been published that nano–TiO_2_ and nano–SiO_2_ added to special-effect inks can have an influence on the surface and mechanical properties of the deposited films and that they have a significant effect on the photocatalytic activity of coatings and UV protection [9,10]. Since titanium dioxide occurs in various polymorphs, anatase and rutile are the most commonly used polymorphs in various coatings. Hanaor et al. [11] showed that the presence of one or both of these titanium dioxide phases affects the photocatalytic performance of the material. In addition, it has been published that nano–TiO_2_ can have a positive effect on other properties of various materials. For example, it has been shown that the addition of rutile titanium oxide nanoparticles positively affects the mechanical properties, thermal stability, weathering resistance, and antibacterial properties of styrene-acrylic-polyurethane coatings [12]. Furthermore, the positive effect of adding TiO_2_ anatase to paints containing pigment TiO_2_ has been published as well. Al-Kattan et al. [13] concluded that paints containing nano–TiO_2_ release limited amounts of titanium into the environment and represent an opportunity for hazard reduction. The addition of TiO_2_ and ZnO nanoparticles to paints was studied in order to determine their anticorrosive, antibacterial, and self-cleaning effects on different types of materials, such as carbon steel sheets, wood sheets, and aluminum [2]. It has been shown that a low concentration of nanoparticles has a beneficial effect on solid surfaces in construction and textile applications. Furthermore, it has been previously published that ZnO nanoparticles can be used to improve the radiation-shielding properties of epoxy resin composites [14]. Additionally, ZnO nanofillers have been used to provide a highly protective layer together with polyvinyl alcohol as a UV blocker and used as optimized films for packaging applications [15].

Due to the wide range of packaging applications and the variety of materials that can be used for packaging, complex and systematic research of the interactions between the materials in contact is of great importance. Furthermore, different printing techniques are used in the packaging industry today: flexographic printing, offset printing, gravure printing, and others. Each printing technique has different requirements, uses different materials and systems for transferring the ink to the substrate, and the interactions between the materials involved in each printing process are different [16,17]. These observations point out that each of the aforementioned printing processes is extremely demanding, as all production requirements, as well as the functional properties of the packaging product, must be reconciled. For this reason, the study of material interaction in these processes is of great complexity, since the surface properties of the materials involved, and their control and stability, play a crucial role in all printing applications. 

In this study, the surface, mechanical, optical, and color properties of ink films with added nanoparticles printed on paperboard substrates were observed. Specifically, the goal was to use a surface modification process to adjust the surface free energy and adhesion properties of applied ink films. Surface modification techniques are sometimes applied to different substrates to adjust properties and control the interaction between materials. In printing processes, various surface modification techniques can be applied, such as plasma and corona treatment, laser alloying of the surface, vapor deposition, thermal diffusion, and others [18,19,20,21,22,23,24]. 

In this research surface modification was achieved through additional exposure of the observed ink films to xenon radiation. The stability and surface properties of applied ink film are extremely important for the printed information on the packaging products and are of great significance if the printed ink films are used as a primer for other coatings or varnishes. To our knowledge, this type of research has not been sufficiently published, and the presented results will increase the knowledge about the possibility of using inks with added nanomaterials in packaging processes.

## 2. Materials and Methods

### 2.1. Preparation and Deposition of the Ink Films

Nanoscale anatase TiO_2_ (Alfa Aesar GmbH&Co. KG, Haverhill, MA, USA), rutile TiO_2_ (Alfa Aesar GmbH&Co. KG, Haverhill, MA, USA), and ZnO (Alfa Aesar GmbH&Co. KG, Haverhill, MA, USA) were added to the base ink material in different amounts. The materials used for deposition were prepared by homogenizing the nanomaterials with commercial UV-curable water-based ink (Sun Chemical Black Process, Solarflex SINT 46, Parsippany, NJ, USA). UV-curable inks are printing inks that dry by ultraviolet (UV) radiation (wavelength range of 100–380 nm). The tested inks were prepared by mechanically mixing the nanoparticles in a defined amount with the base ink. The mass concentration of nanoparticles added to the base ink was adjusted to 0.5%, 1%, and 1.5%. A total of ten (10) samples of different inks, nine (9) inks with added nanoparticles and one (1) sample of ink without nanoparticles, were prepared. The mixture was homogenized with an ultrasonic disperser (UP100H Hielscher, Hielscher Ultrasonics GmbH, Teltow, Germany) for 2 min at 100% device amplitude. The properties of the used nanoparticles are listed in Table 1.

The prepared inks were deposited on uncoated and coated paperboards by a laboratory flexographic testing device (IGT F1, IGT Testing Systems, Almere, The Netherlands) [25]. The printed samples were dried in a UV dryer (Aktiprint L, Technigraf GmbH, Grävenwiesbach, Germany) at a speed of 4 m/s in two passes. After the samples underwent a 24 h stabilization period, the measurement and surface modification treatment were carried out. 

The main properties of used paperboards are listed in Table 2 with included standard deviation (SD). Samples were conditioned at 50 ± 2% relative humidity and a temperature of 23 ± 1 °C prior to measurements (ISO 187). Caliper was determined using a thickness gauge DGTB001 (Enrico Toniolo S.r.l., Milan, Italy) and measured on twenty (20) samples for each paperboard according to ISO 534:2011. Grammage was measured on ten (10) samples according to ISO 536. Before the deposition process, the paperboards were conditioned at a temperature of 23 ± 1 °C and 50 ± 2% relative humidity.

**Figure 1:**
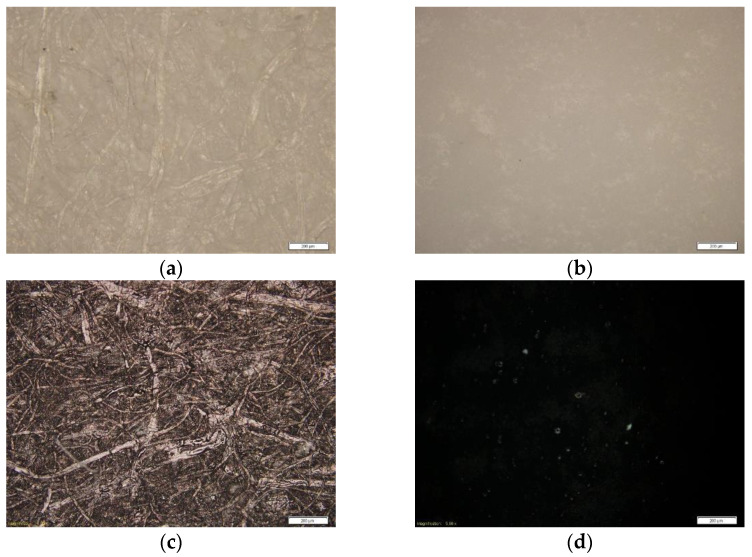
Microscopic images of the samples: (**a**) uncoated; (**b**) coated; (**c**) uncoated paperboard with ink film; (**d**) coated paperboard with ink film (mag. 50×).

In order to observe the differences in the surface morphology of paperboards, the micrographs of the samples are shown in Figure 1. Figure 1a,b presents the morphology of paperboards without ink, and Figure 1c,d presents it with deposited unmodified printing ink. Micrographs of samples were obtained using an Olympus BX51 microscope (Olympus Corporation, Tokyo, Japan). The difference in the surface texture of uncoated and coated paperboards can be clearly seen in Figure 1. The surface of the uncoated paper is interspersed with cellulose fibers arranged in different directions, indicating an irregular structure of the sample. Figure 1c shows the surface of deposited unmodified ink film, with ink particles embedded in the paperboard structure. Figure 1b shows coated paperboard; its structure is smooth, without visible irregularities and cellulose fibers. The printed ink film applied to the coated paperboard is flat and uniformly structured without any irregularities, as can be seen in Figure 1d.

### 2.2. Surface Modification

Surface modification was performed on the samples after the prints were made and after a stabilization period of 24 h. Surface modification was conducted by exposing the samples to xenon radiation in a Solarbox 1500e test chamber (CO.FO.ME.GRA., Milano, Italy). The xenon light exposure simulates realistic natural outdoor weathering conditions; an artificial daylight indoor filter (S208/S408) was used. Irradiation was set at 550 W/m^2^ at a temperature of 50 °C and a relative humidity of 65%. The equipment was set according to the standard ISO 4892–2. The samples were exposed to radiation for 12 and 24 h.

### 2.3. Methods

#### 2.3.1. FTIR Spectroscopy

FTIR-ATR analysis was performed in order to determine the changes occurring in the deposited unmodified inks and nanoparticle-modified inks before and after the surface modification. The IRAffinity–1 FTIR Spectrophotometer (Shimadzu, Kyoto, Japan) was used, crystal type: ZnSe (index of refraction 2.4), number of scans: 15, resolution: 4 cm^−1^). IR spectra were recorded in the spectral range between 3000 and 600 cm^−1^.

#### 2.3.2. Surface Free Energy and Adhesion 

The adhesion of different ink films plays an important role in many processes in the packaging. To ensure optimum stability of the ink films, the adhesion between the applied inks and paperboard must be optimized. In order to define the adhesion between the materials, surface free energies (SFE) of observed materials in contact, in the “paperboard-ink film” system, were calculated. By knowing the SFE of materials, the important surface properties of the deposited ink films and substrates can be evaluated and the interactions between the materials can be determined. These properties are especially important during the ageing process, as photochemical degradation can have an impact on changing the physical and optical properties of the ink films [9,16,26].

In this research the Owens, Wendt, Rabel, and Kaelble (OWRK) method was used for the calculation of the surface free energy [27,28,29,30,31,32]. It is a standard method for defining the surface free energy of a solid surface developed from Young’s equation. It defines the relationship between the contact angle of several probe liquids with known surface tension, the interfacial tension between a liquid and a solid surface, and the surface free energy of the solid. For calculation of the SFE, the following equation was used Equation (1):

(1) $$\gamma_{sv}=\gamma_{sl}+\gamma_{lv}\;cos\theta$$

where *γ_sv_* is the SFE of the solid surface, *γ_sl_* is the solid–liquid interfacial energy, *γ_lv_* is the surface tension of the liquid, and *θ* represents the contact angle of liquid applied on the solid surface. The OWRK method is based on the two-component model to separate the interfacial tension according to the underlying interactions between molecules. The two-component model defines the polar and dispersive interactions between the materials, and the total surface energy of the solid surface is the sum of the two parts. In this work, three probe liquids with known surface tension (demineralized water, diiodomethane, and glycerol) were applied to the samples. The total, dispersive, and polar surface tension components of the probe liquids were, respectively, diiodomethane—50.8, 50.8, and 0; glycerol—64.0, 34.0, and 30.0; and water—72.8, 21.8, and 51.0, all expressed in mJ/m^2^ [28]. Ten drops of each liquid, with 1 μL volume, were applied at different positions on paperboards without ink and on surfaces with deposited ink films. Contact angles were measured using the Sessile drop method, and the average values were used for the calculation of surface free energies. All measurements of the contact angles were performed at 0.4 s after the drop had touched the sample. Surface free energy and contact angles on samples were analyzed using the Data Physics OCA 30 goniometer (DataPhysics Instruments GmbH, Filderstadt, Germany).

Furthermore, in order to calculate the adhesion between the materials, three parameters were observed: interfacial tension (*γ*_12_), work of adhesion (*W*_12_), and wetting coefficient (*S*_12_) [28]. In order to acquire information about the adhesion performance, all three parameters were considered. According to Israelachvili [28], for good adhesion, *W*_12_ should be as high as possible, *γ*_12_ should be close to zero, and *S*_12_ should be positive or equal to zero. 

#### 2.3.3. Rub Resistance

Rubbing of the deposited ink films on packaging can occur during handling and different production stages, shipping, and storage, or by the end user. Damage to the surface of deposited ink films can cause significant deterioration of the legibility of the printed information and degradation of the colour appearance. In this work, the RT4 Hanatek Rub and Abrasion Tester (Rhopoint Instruments GmbH, Gaukönigshofen, Germany) was used to test the durability of unmodified and modified films on observed paperboards. The instrument works by the method of rubbing a deposited sample against a reference paper under defined conditions. In this work, paperboards with a diameter of 50 mm with deposited inks were cut and placed against the standard offset paper with a diameter of 115 mm (grammage 80 g/m^2^). 

The tests were performed according to the standard BS 3110, method 2 ‘Methods for measuring the rub resistance of print—rotary method’. The pressure was set at 2 psi (13.8 kPa), and 50 rotations were performed for each sample. The specimens were then visually evaluated and assigned by grades from 1 to 5. The sample with the highest resistance to rubbing (imperceptible rubbing) was scored 1, and the sample with the lowest resistance (very pronounced rubbing) was scored 5. Tests were performed on unmodified and modified samples in order to determine the effect of nanoparticles on the stability of printing ink films on paperboard substrates.

#### 2.3.4. Resistance to Bending

Resistance to bending is a mechanical property of paperboards that is related to the usability and durability of packaged products and in end-use situations. In this work, resistance to bending was measured using the Lorentzen & Wettre bending tester (ABB AB /Lorentzen & Wettre, Zurich, Switzerland). It can be used to determine the bending resistance of paper and paperboard by measuring the force needed to bend a sample to a predetermined angle, according to the ISO 2493–2:2020 Paper and board—Determination of resistance to bending—Part 2: Taber-type tester. The resistance to bending of the samples was measured in order to analyze the influence of the addition of nanoparticles in inks on the bending resistance of the samples. Furthermore, it was used to define the influence of the surface modification on the mechanical properties of the printed ink films. The resistance to bending was measured three times for each sample at an angle of 7.5°. The bending angle was defined according to the valid standard, taking into account the properties of the tested samples. It was assumed that the optional value of the bending angle (15°), as purposed by the standard, could damage the ink film and lead to “creep” or cracking of the bended surface of the printed ink films.

#### 2.3.5. Color Properties of Deposited Ink Films

The color properties of ink films were measured using spectrophotometer X-Rite Gretag Machbeth Eye One Pro (X-Rite, Grand Rapids, MI, USA). Measurements were performed to evaluate the color properties of the applied unmodified and modified ink films. Additionally, measured values were used to evaluate the influence of surface modification on the color stability of deposited ink films. The results of the spectral reflectance were used to detect the color density (*D*) and the color characteristics in the CIE L*a*b* color space [33]. Color density is a value calculated from the amount of light that is reflected from the printing substrate (paper, film, etc.) and the applied ink. CIE L*a*b* is a three-dimensional color space defined by the International Commission on Illumination (CIE). It covers the entire visual color range of human color perception. The L* coordinate represents the lightness of the color sample, a* is the position relative to the green–red opponent colors, and b* axis represents the blue–yellow opponents. Before the measurement, the spectrophotometer was calibrated to a reference white, with Standard Illuminant D50, observer angle 2°.

## 3. Results and Discussion

### 3.1. Measurements Performed on Unmodified Ink Films 

#### 3.1.1. FTIR–ATR Analysis

**Figure 2:**
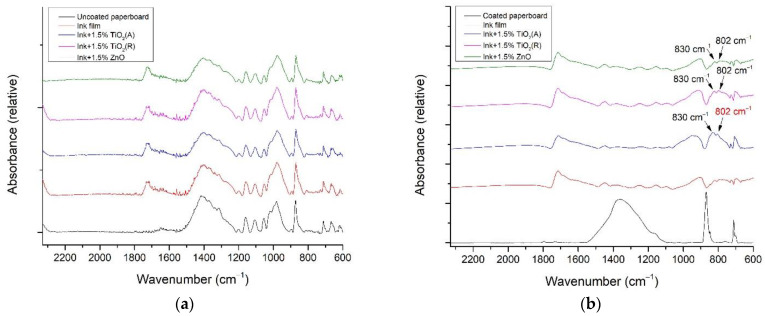
FTIR–ATR spectra of paperboards with deposited ink films and the addition of 1.5% of TiO_2_ (A), TiO_2_ (R), and ZnO nanoparticles; (**a**) uncoated paperboard and (**b**) coated paperboard.

The results of FTIR–ATR analysis of uncoated and coated paperboards with deposited ink films and addition of 1.5% of nanoparticles are shown in Figure 2, since no changes were detected when lower concentrations of the nanoparticles were added to the ink. The main objective of the FTIR–ATR analysis was to identify the changes in the functional groups in the modified ink films, which could indicate the changes in the surface of the inks caused by the addition of TiO_2_ (A)—anatase, TiO_2_ (R)—rutile, and ZnO nanoparticles.

It is visible in Figure 2 that nanoparticles did not affect the vibrations of bonds/groups in the FTIR–ATR spectrum, except for 1.5% of TiO_2_ (A) on coated paperboard (Figure 2b), where after the addition of 1.5% TiO_2_ (A), a peak at 802 cm^−1^ appears. This could be the consequence of the drying process of deposited inks caused by UV radiation, where the twisting vibration of CH_2_=CH– appears [34]. It is possible that the addition of anatase TiO_2_ inhibits UV radiation, because the absorbance increases at the peak, which means that the concentration of 1.5% TiO_2_ (A) is too high and interferes with the drying of the ink. Such a result is expected, since TiO_2_ (A) is more reactive to UV than rutile TiO_2_ [35], which is later visible by the slight reduction of the polar phase of SFE after the addition of 1.5% anatase TiO_2_. This change is not visible on uncoated paperboard, probably due to the irregularities in the surface structure visible in Figure 1a,c.

#### 3.1.2. Surface Free Energy and Adhesion Properties

**Figure 3:**
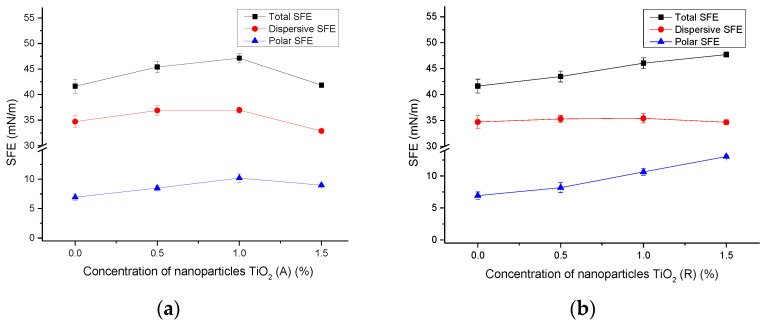

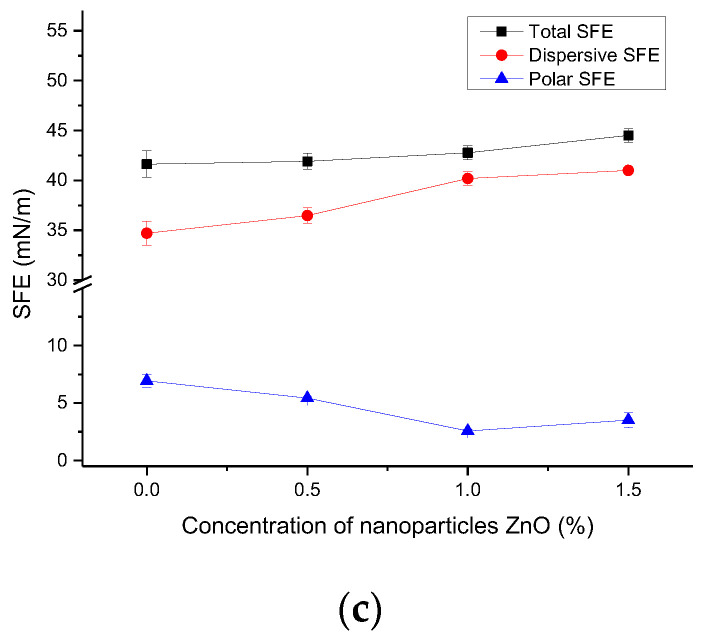
SFE of uncoated paperboards with deposited ink films without and with the addition of nanoparticles; (**a**) TiO_2_ (A), (**b**) TiO_2_ (R), and (**c**) ZnO.

**Figure 4:**
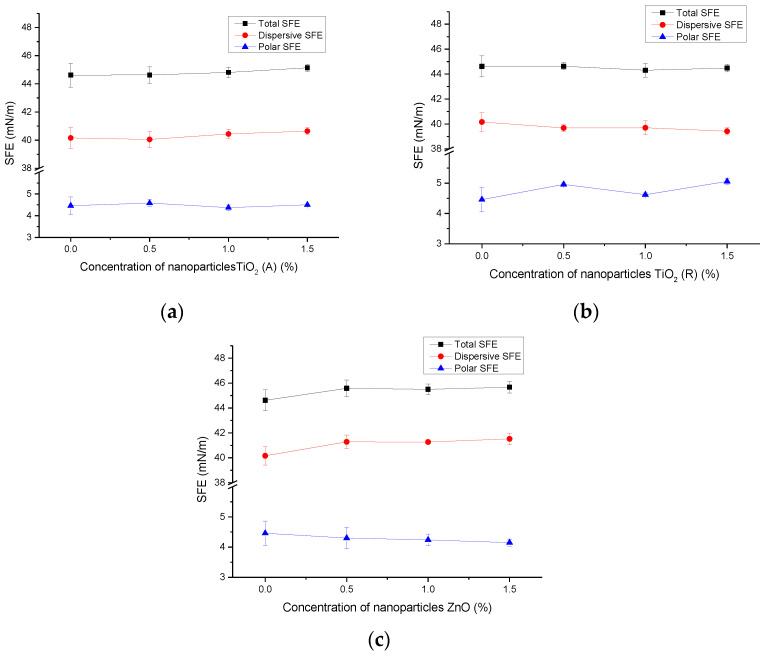
SFE of coated paperboards with deposited ink films without, and with the addition of nanoparticles; (**a**) TiO_2_ (A), (**b**) TiO_2_ (R), and (**c**) ZnO.

Results of the surface free energy measured on paperboard substrates are presented in Figure 3 and Figure 4, and they indicated the difference in the observed surfaces. The total SFE measured on uncoated paperboard equals 26.55 mN/m, with 26.42 mN/m of the dispersive part of SFE and 0.12 mN/m of the polar part of SFE. On the other hand, the total SFE of the coated paperboard equals 24.2 mN/m, with 15.76 mN/m of the dispersive part and 8.44 mN/m of the polar part. It can be said that the total SFE of uncoated paperboard is higher than that of coated, and that its surface has a more-pronounced dispersive component than coated paperboard.

Figure 3 presents the results of surface free energy calculations on deposited ink films without and with the addition of nanoparticles on uncoated paperboards. One can see that different nanoparticles cause similar changes in the SFE on uncoated paperboard samples (Figure 3a–c). It can clearly be seen that due to the addition of nanoparticles, the SFE of the film surfaces have been slightly increased. The total SFE for all samples without nanoparticles is 41.63 mN/m, and with the addition of TiO_2_ (A) SFE is increased to a maximum value of 43.38 mN/m (with 0.5% TiO_2_). However, with the addition of 1.5% TiO_2_, this slightly decreases to 41.84 mN/m, which is still higher than the initial SFE without nanoparticles. The reason for decrease of SFE is probably lies with the appearance of agglomerates in the deposited ink film specific to materials with added nano–TiO_2_ [36]. With the addition of rutile TiO_2_ nanoparticles, the SFE increases gradually with the proportion of nanoparticles, and its highest value was calculated for 1.5% (47.7 mN/m). ZnO nanoparticles also cause a slight and gradual increase in SPE to 44.51 mN/m. Although the total SFE of the ink films changes to a small extent, a more significant change is visible in the polar phase of samples. One can see that the addition of different amounts of anatase TiO_2_ and rutile TiO_2_ causes an increase in the polar SFE phase of samples, and that by the addition of ZnO nanoparticles the polar phase is decreased. Obviously, the high hydrophilicity of nano–TiO_2_ leads to improvement in the hydrophilic character of the observed ink films, which corresponds to the studies published before [9,37]. 

The measured range of SFE values obtained by adding nanoparticles at different concentrations to the base ink is useful for optimizing and adjusting the surface properties of the deposited ink films. Moreover, the SFE of the prepared ink can be tuned to the surface properties of different substrates, which affects the optimal adhesion between the deposited films and the substrates. In addition, it was found that the polar component of SFE is low in most of the deposited ink films and that it can be increased by the addition of titanium dioxide nanoparticles at certain concentrations. These results may also be useful when an ink with added nanoparticles is used as a primer for other coatings or varnishes.

Figure 4 presents the results of the surface free energy of ink films without and with the addition of nanoparticles on coated paperboard samples. It is visible that the total SFE is slightly increased with the addition of higher concentrations of TiO_2_ (A) and ZnO nanoparticles. On samples of 1.5% TiO_2_ (A) and 1.5% ZnO, the SFE is at its maximum value. With the addition of TiO_2_ (R), the SFE is slightly decreased. Overall, one can say that the changes in total SFE are not as pronounced as those measured on an uncoated paperboard surface. A slight difference is detected in the polar component of SFE which has been decreased with the addition of TiO_2_ (A) and ZnO nanoparticles. The polar component of SFE is increased by the addition of TiO_2_ (R) in deposited ink film. 

It can be said that the addition of nanoparticles in the prepared inks caused different surface effects on different paperboards. Obviously, the differences in the surface free energies of paperboards have a significant impact on the surface properties of the applied ink films. The changes in surface polarity and total SFE were more pronounced on the uncoated paperboard surface in comparison to coated one, suggesting that the interactions between the modified inks and paperboards are different.

Table 3 and Table 4 show the results of calculating the adhesion parameters on uncoated and coated paperboard samples. The conditions for optimum adhesion are given by the value of the maximum thermodynamic work of adhesion (*W*_12_ = max), the positive value of the spreading coefficient (*S*_12_), and the minimum value of the interfacial tension (*γ*_12_). That is, in order to achieve optimum adhesion, the interfacial tension should be minimal (tending towards zero) and the optimum spreading coefficient should be as close as possible to zero or a positive value. It can be seen from results that the interfacial SFE has the lowest values in the systems with the addition of anatase TiO_2_ nanoparticles in the ink on uncoated paperboard (Table 3). 

The approximate values of interfacial tension were calculated in systems with the addition of nano–ZnO, while the other values of interfacial tension were increased with the addition of rutile TiO_2_, indicating weaker adhesion. As for the work of adhesion, an increase in the value (better adhesion) can be seen in all the observed systems with added nanoparticles. The values of the wetting coefficient are positive, indicating good adhesion between the observed materials. The values of the adhesion parameters of ink films on coated paperboards showed that the addition of rutile TiO_2_ has a better effect on adhesion compared to other nanoparticles.

#### 3.1.3. Mechanical Properties 

Table 5 shows the results of the visual assessment of rub resistance of deposited ink films, which were evaluated according to the following criteria: 1—imperceptible rubbing, 2—small signs of rubbing, 3—visible rubbing, 4—pronounced rubbing, 5—very pronounced rubbing. As can be seen, the addition of nanoparticles in the ink has mostly an unfavorable effect on the rubbing, because the visual assessment values on both printing substrates are between 3 and 4 (visible and pronounced rubbing of the ink films); only the samples without nanoparticles have a score of 1 (imperceptible rubbing of the prints). A positive effect on rub resistance was detected for uncoated and coated paperboards with the addition of 1% TiO_2_ (A) and 1.5% ZnO in the ink films, showing the criteria 1 and 2, imperceptible rubbing and small signs of rubbing, and on ink film with the addition of 1.5% TiO_2_ (R) on coated paper.

**Figure 5:**
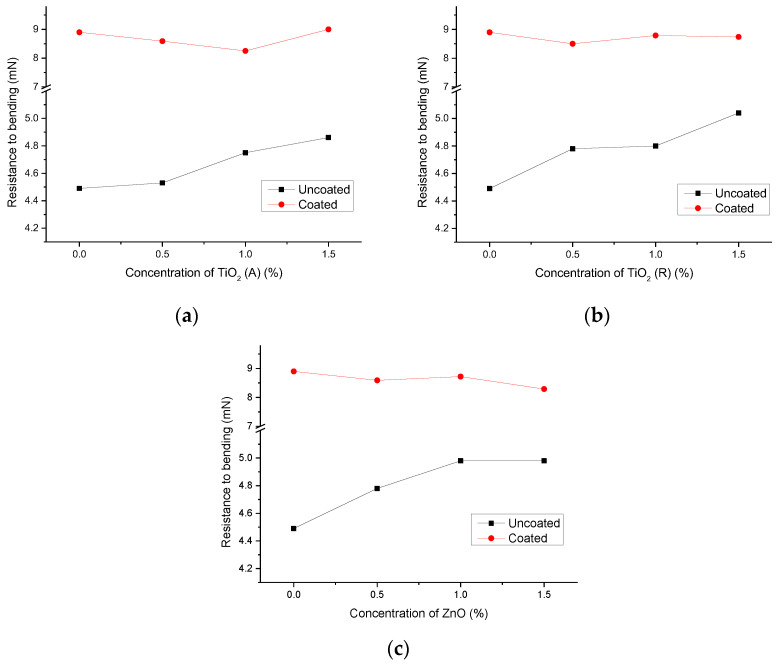
Resistance to bending of uncoated and coated paperboards with deposited thin films and the addition of nanoparticles: (**a**) TiO_2_ (A); (**b**) TiO_2_ (R); (**c**) ZnO.

The results of the resistance to bending of the observed samples are presented in Figure 5. Paperboards, without the deposited ink film, have a resistance of 2.19 mN and 3.67 mN, on uncoated and coated paperboards, respectively. One can see that the addition of nanoparticles significantly affects the resistance to bending of uncoated paperboard, where the measured values are increased due to the addition of nanoparticles. On the other hand, the effect of the addition of nanoparticles on the resistance to bending of the deposited inks is insignificant on coated paperboard. It can be assumed that the surface structure of the uncoated paperboard enabled the filling of the surface’s unevenness with deposited inks and nanoparticles, and consequently caused an increase in the bending resistance of samples. In the case of coated paper, the addition of nanoparticles in the base ink has a slight, almost insignificant influence on the resistance to bending of the samples.

#### 3.1.4. Color Measurement

The color measurement of the samples expressed by the CIE L*a*b* coordinates and the optical density (*D*) results are shown in Table 6. One can see that the addition of nanoparticles has no significant effect on the CIE L*a*b* coordinates of the deposited ink films. Moreover, the addition of nanoparticles has no significant effect on the lightness (L*) of the observed inks.

It can be noticed that the lightness coordinate of ink films on coated paperboards has lower values than the lightness measured on uncoated paperboards. These results can be explained by the different surface structures of the paperboards, which obviously affect the optical properties of the deposited inks. The surface structure of inks applied to uncoated paper is not homogeneous but rather interspersed with cellulose fibers, which are visible in the structure of the paperboard (Figure 1c). Even though the UV-curable inks dry instantly under exposure to UV radiation, the printing ink obviously partially penetrates the structure of the uncoated paperboard, which causes an uneven surface structure of the applied ink film [16,38,39]. In order to completely cover the surface structure of the uncoated substrate, a larger amount of ink must be used compared to a coated surface. In this study, the ink samples were applied in a single pass through the printing device. Compared to uncoated paperboard, coated paperboard has a coating material that is able to absorb the ink and form a homogeneous and smooth ink film on the surface. The applied film completely covers the surface of the paperboard (Figure 1d). The optical density results show that the inks without and with nanoparticles have similar *D* values. *D* varies between 1.734 and 1.836 on uncoated paperboard and between 2.9 and 2.926 on coated paperboard. The differences in the density of printing inks applied to coated and uncoated surfaces are the result of the different surface structures of the initial paperboards.

### 3.2. Measurements Performed on Modified Ink Films 

Surface modification by xenon lamp was carried out with the aim of improving the surface and adhesion properties of deposited ink films on paperboard. Additionally, it was determined whether the added nanoparticles improve the photostability of the deposited films, their mechanical properties, and their influence on the optical appearance of the color. The analysis was performed after the surface modification was conducted for 12 and 24 h on paperboard substrates without ink, on substrates with deposited ink films without and with the addition of 1% nanoparticles. 

#### 3.2.1. FTIR–ATR Analysis

**Figure 6:**
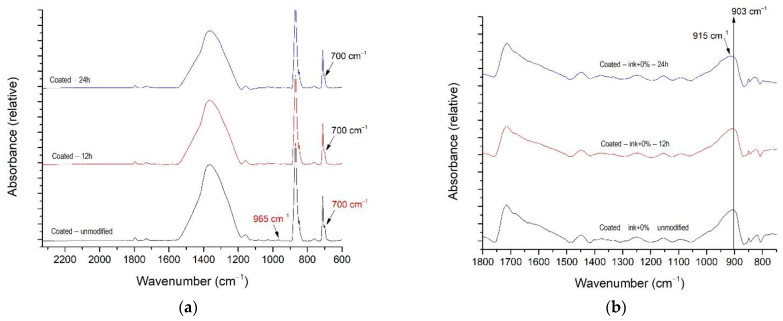
FTIR–ATR spectra of unmodified and modified samples; (**a**) coated paperboard and (**b**) printed ink on the coated paperboard without nanoparticles.

Figure 6a shows the FTIR–ATR spectra of unmodified and modified coated paperboard substrates with and without the ink film (Figure 6a and Figure 6b, respectively). The surface modification by xenon radiation did not cause any significant change in the uncoated paperboard. A small difference can be seen in the spectra of the modified coated paperboard samples. After the coated paperboard was exposed to xenon radiation for 12 and 24 h, the peak at 965 cm^−1^ was no longer visible (Figure 6a). Obviously, the modification process caused the changes in the paperboard coating and led to additional crosslinking and/or degradation in the coating structure. 

Most likely, the observed change in the spectrum can be attributed to the CH_2_ vibration [40,41]. In addition, a peak at 700 cm^−1^ was not detected in coated paperboard after surface modification. It was located in the fingerprint region and could be attributed to the out-of-plane C–H vibration that occurs in latex, which is often used as a binder in paper coatings. Latex particles have the role of binding the pigment particles and adhering them to the base material. They also affect the rheology of the coating, the properties of the coated paperboard, and its printability [42,43]. 

Figure 6b shows the FTIR–ATR spectra of the unmodified and modified samples of the ink film without nanoparticles applied to the coated substrate. It can be seen that the peak at 903 cm^−1^ shifted to 915 cm^−1^, with the whole band increasing after the modification process. This is probably a consequence of the change in the vibrations of the epoxy group, which is normally present in the composition of UV-curable inks [44,45].

**Figure 7:**
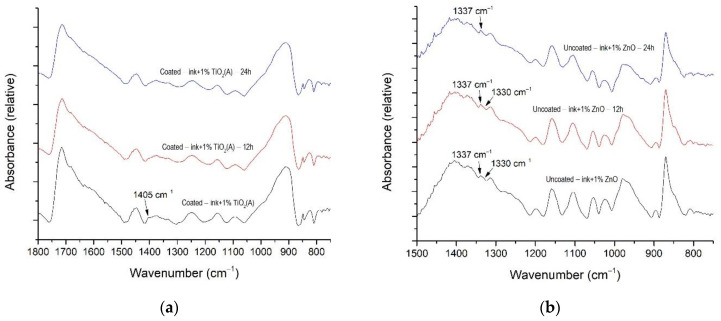
FTIR-ATR spectra of unmodified and modified ink films with addition of nanoparticles: (**a**) TiO_2_ (A) on coated paperboard and (**b**) ZnO on uncoated paperboard.

The chosen FTIR–ATR spectra of unmodified and modified samples with the addition of nanoparticles are shown in Figure 7. Only the spectra where the first exposure-induced changes occurred are presented. 

On coated paperboard, a change was observed for the ink film with 1% of TiO_2_ (A) nanoparticles at the peak 1405 cm^−1^ after 12 h of exposure (Figure 7a). Probably the composition of the deposited ink changed due to the disappearance of the naphthalene ring, as already shown in [46,47]. In addition, changes in the ink film due to the addition of ZnO nanoparticles were detected only on the uncoated paperboard (Figure 7b). The band between 1330 cm^−1^ and 1337 cm^−1^ changed the shape with the surface modification. This change is probably due to changes in the diisocyanate group in the composition of the ink [44,48,49]. The analysis of the FTIR–ATR spectra of the ink films with TiO_2_ (R) showed that no changes were detected on the surface of the printed ink films. Therefore, it can be said that rutile TiO_2_ best inhibits the xenon radiation modification-induced changes in the observed inks on both paperboards. These results are in line with previous studies where rutile TiO_2_ had improved the UV protection of various coatings [12,50,51].

#### 3.2.2. Surface Free Energy and Adhesion Properties

The results of the calculated SFEs of ink films applied to uncoated and coated paperboards and additionally modified are shown in Table 7. It can be seen that the total SFE (*γ*^total^) of the ink films on both substrates was increased by the addition of 1% nanoparticles (details can be seen in Figure 4). Surface modification caused some additional differences in the SFEs. It can be seen that the total SFE decreased for all observed samples. Obviously, the exposure to xenon radiation caused additional changes in the ink film layer, especially in the polar component of the SFE (*γ*^P^), which was decreased on all ink films printed on uncoated paperboard. It can be seen that the decrease is almost the same for all modified inks on uncoated paperboard. 

SFEs calculated on ink films on coated paperboard are quite different. The total SFE decreased with surface modification on all samples, but unlike the results on uncoated paperboard, the polar component of the SFE was increased. These results are consistent with the results shown in Figure 2 (unmodified samples). 

The adhesion parameters between the substrates and ink films on the modified samples were calculated to obtain information about the changes in the interactions between the applied inks and paperboards caused by xenon radiation. Since adhesion depends upon the intermolecular physical and chemical bonds between the ink and the substrate, the strength of the interactions affects the stability of the ink applied to the substrate [52]. 

**Figure 8:**
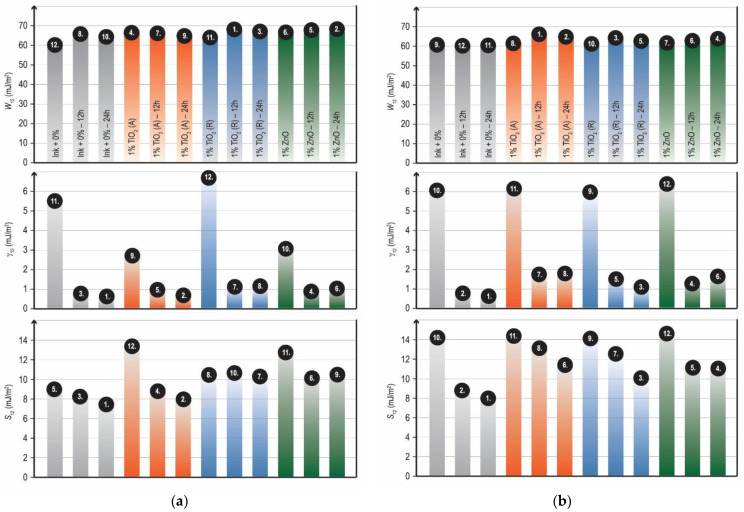
Adhesion parameters of the unmodified and modified ink films without and with addition of nanoparticles on (**a**) uncoated and (**b**) coated paperboards.

The results obtained from samples before and after surface modification are shown in Figure 8. Some differences in the adhesion parameters were observed. By analyzing the SFE of interphase (*γ*_12_) between the observed inks on both substrates, significant differences are visible between the unmodified and modified samples. 

The UV inks used in this research consist of monomers, prepolymers/oligomers, pigments, additives, and photo initiators. During UV irradiation, polymerization takes place in the structure of the ink, resulting in a crosslinked structure, i.e., a firm ink film on the substrate [47]. With additional surface modification, the ink structure apparently changes uniformly on both paperboards and forms an ink film with lower values of the SFE of the interface, indicating better adhesion of the observed films. 

By analyzing the results of the work of adhesion (*W*_12_), which should be as high as possible to indicate good adhesion, one can see that there are some differences between the “ink–substrate” pairs. For modified inks with rutile TiO_2_ and ZnO nanoparticles applied to uncoated paperboard, the work of adhesion is higher, indicating better adhesion after 12 and 24 h of surface modification compared to coated samples. On coated paperboard, the work of adhesion has the highest values for samples with the addition of anatase TiO_2_ in the ink film. When evaluating the results of the wetting coefficient (*S*_12_), all samples have a positive value, indicating good adhesion of the samples after surface modification.

#### 3.2.3. Mechanical Properties

An important property of all printed ink films is a good bond with the substrates so that they do not detach from the surface in end-use situations. After the samples were subjected to the surface modification process, the rub resistance test was performed. Table 8 shows the results of the visual evaluation of the unmodified and modified ink films. It can be said that the nanoparticles have a positive effect on the rub resistance of the samples after the surface modification. The results obtained on uncoated paperboard showed that the addition of rutile titanium oxide and ZnO nanoparticles has the greatest effect on the rub resistance of the ink films. After modification of 24 h, these results were rated as 1, i.e., an imperceptible rubbing of the prints. In addition, it can be seen that the addition of rutile titanium oxide and ZnO nanoparticles in ink applied to coated paperboard greatly improves the rub resistance of ink films. It can be seen that initial results obtained for unmodified samples (4—pronounced rubbing, 5—very pronounced rubbing) have changed to a score of 1, imperceptible rubbing. One can conclude that the additional surface modification by xenon radiation improved the rub resistance of the ink film samples with added rutile nano–TiO_2_ and ZnO. 

**Figure 9:**
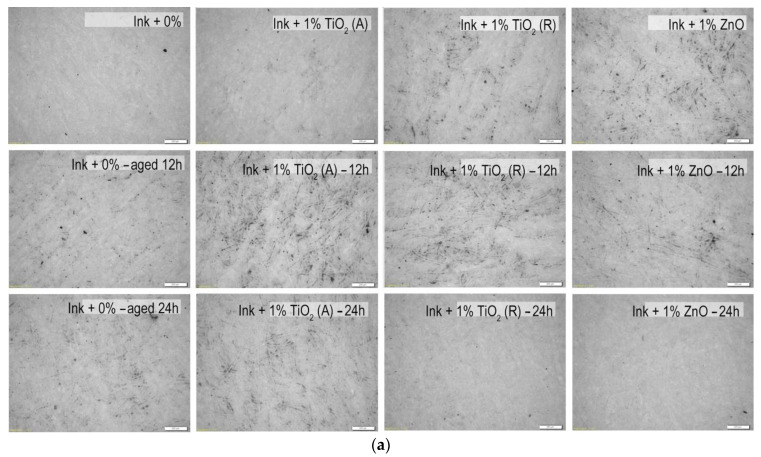

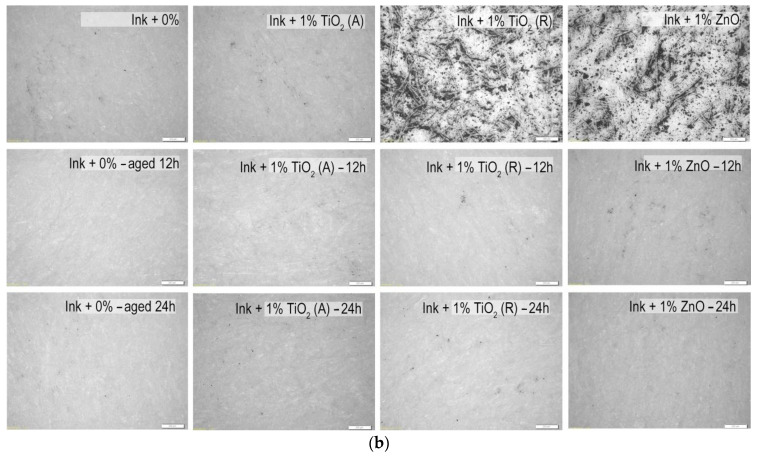
Microscopic images of the samples after performing the rub resistance test on unmodified and modified ink films on (**a**) uncoated and (**b**) coated paperboards (mag. 50×).

The microscopic images of the samples after performing the rub resistance test are shown in Figure 9. The images show the positive effect of the surface modification and nanoparticles on resistance to rubbing of the observed samples. 

It can be seen that all nanoparticles caused similar rub resistance. Rutile TiO_2_ and ZnO nanoparticles showed significant improvement in rub resistance after 24 h of exposure on an uncoated paperboard substrate (Figure 9a). Furthermore, one can see that after 12 h of surface modification significant improvement in rub resistance was detected on coated paperboard (Figure 9b). Overall, those results are in correspondence with the adhesion properties of the same samples, because they have the highest values of thermodynamic work of adhesion (*W*_12_), indicating good adhesion between the modified materials (Figure 8).

**Figure 10:**
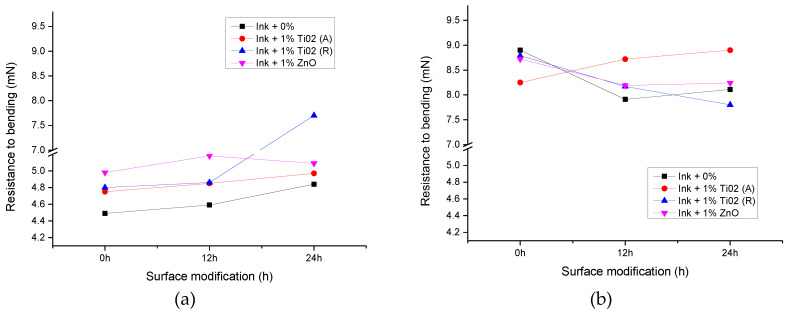
The resistance to bending of unmodified and modified samples with TiO_2_ (A), TiO_2_ (R), and ZnO nanoparticles; (**a**) uncoated and (**b**) coated paperboard.

Resistance-to-bending results are presented in Figure 10. It can be seen that resistance to bending was improved in samples by the xenon radiation.

Resistance-to-bending tests performed on samples showed a positive effect of surface modification on uncoated paperboard with the addition of all observed nanoparticles (Figure 10a). The results of the measurements on coated substrates showed that the addition of anatase TiO_2_ can improve the bending resistance of paperboard (Figure 10b). Other nanoparticles, especially rutile TiO_2_, did not have a positive effect on bending resistance.

#### 3.2.4. Color Measurement

**Figure 11:**
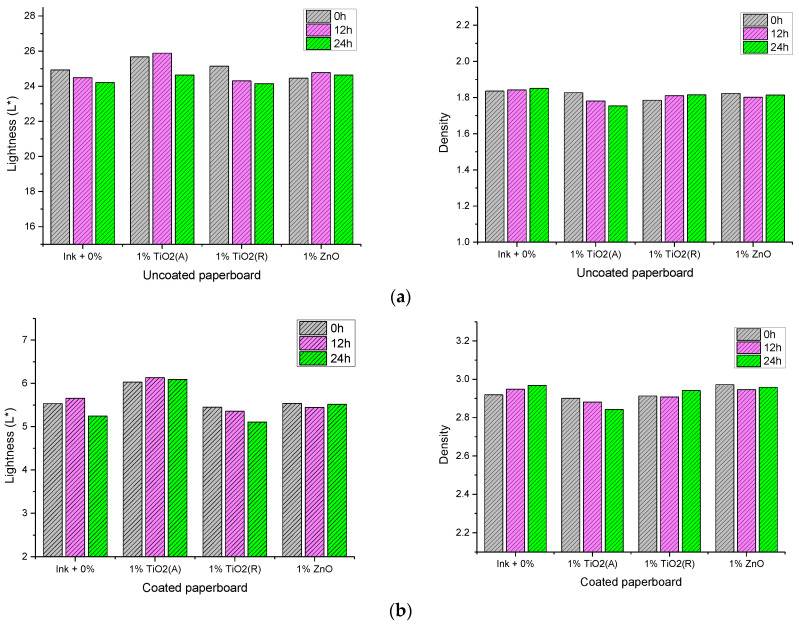
Results of the CIE L* values and color density of unmodified and modified ink films; (**a**) uncoated and (**b**) coated paperboard.

The results of the color measurement are shown in Figure 11. The results of the CIE lightness (L*) values and color density (*D*) of the unmodified and modified samples with the addition of 1% nanoparticles are presented. It can be seen that the addition of nanoparticles and surface modification have a slight influence on the lightness and optical density of deposited ink films. A detail analysis of results showed that the addition of anatase TiO_2_ and surface modification caused an increase in the CIE lightness values of uncoated and coated deposited ink films. Since the lightness value (L*) defines black at 0 and white at 100, one can say that the addition of anatase TiO_2_ and the surface modification of ink films did not improve the color properties. On the other hand, other nanoparticles, especially rutile TiO_2_, showed a positive effect on lightness from surface modification. It can be seen that the lightness of both paperboards is decreased by surface modification, meaning that the printed black did not become lighter in comparison to unmodified ink film. Similar results have been observed with density value. One can see that the surface modification of samples with the addition of rutile TiO_2_ and ZnO nanoparticles by xenon radiation showed a slight improvement in the density of printed ink films. It can be concluded that the addition of nanomaterials (rutile TiO_2_ and ZnO) in the base ink and surface modification have a positive effect on the color properties of the printed ink films. Furthermore, similar results were previously published where those nanoparticles were used to give an UV protection of various coatings [12,50,51].

## 4. Conclusions

In this research, surface modification of thin ink films with added anatase TiO_2_ and rutile TiO_2_ and ZnO nanoparticles was used as a means to improve the properties of applied ink on uncoated and coated paperboard substrates. Surface modification was performed by additional exposure of the samples to xenon radiation. 

Measurements were performed on unmodified and modified samples. The results of the unmodified samples showed that the addition of nanoparticles in the prepared inks caused different surface effects on the considered paperboards. The changes in surface polarity and total surface free energies were more pronounced on the uncoated paperboard surface than on the coated one, proving that the interactions between the prepared inks and paperboards were different. The values of the adhesion parameters of the Ink films showed that the addition of anatase TiO_2_ to the ink applied to uncoated paperboard and rutile TiO_2_ to the ink applied to coated paperboard had a better effect on adhesion compared to other nanoparticles. The results of the mechanical properties showed that the addition of nanoparticles in the inks caused low sighing and pronounced rubbing of the ink films. The addition of nanoparticles to the inks had a positive effect on resistance to bending, especially for the ink films applied to uncoated paperboards. Color analysis showed that the addition of nanoparticles did not change the optical properties of the ink films.

The measurements performed on the samples after surface modification showed a significant improvement in the surface, adhesion, mechanical, and color properties of the observed samples.

The results of FTIR–ATR measurements performed on the ink films after surface modification showed that xenon radiation did not cause significant changes to the ink films without nanoparticles applied to uncoated paperboard. A slight difference was detected in the films applied to coated paperboard. For the surface modification of films containing nanoparticles, the FTIR–ATR spectra showed that the change for the ink film with 1% TiO_2_ (A) nanoparticles was detected at peak 1405 cm^−1^ after 12 h of the radiation, probably due to the disappearance of the naphthalene ring in the composition of the deposited ink on coated paperboard. In addition, changes in the ink film due to the addition of ZnO nanoparticles were detected only on the uncoated paperboard. The band between 1330 cm^−1^ and 1337 cm^−1^ changed shape with surface modification, probably due to changes in the diisocyanate group in the ink composition. Analysis of the FTIR–ATR spectra of the ink films with TiO_2_ (R) showed that no changes were detected on the surface of the printed ink films, proving that rutile TiO_2_ inhibits xenon radiation best.

The results of the influence of surface modification on the SFEs and adhesion parameters of the deposited ink films showed that the total SFE decreased for all observed samples. Exposure to xenon radiation caused additional changes in the ink film layer, especially in the polar component of the SFE, which was decreased for all ink films deposited on uncoated paperboard. The SFEs calculated on ink films on coated paperboard were quite different. The total SFE decreased with surface modification on all samples, but unlike the results on uncoated paperboard, the polar component of the SFE was increased. These results had a significant effect on the adhesion between the deposited ink films and paperboard substrates. Surface modification caused the same changes on both paperboards and formed an ink film with lower values of SFE of the interface, indicating better adhesion of the observed films. Results of the work of adhesion showed that the surface modification of ink films with rutile TiO_2_ and ZnO nanoparticles on uncoated paperboard improved the adhesion between the materials. In addition, results of the work of adhesion calculated between the ink films with the addition of anatase TiO_2_ and coated paperboard also showed improved adhesion.

The results of the mechanical properties measured on modified ink films showed a significant improvement in the rub resistance of the samples after surface modification. The results obtained on uncoated and coated paperboards showed that the addition of rutile TiO_2_ and ZnO nanoparticles had the greatest effect on the rub resistance of the ink films. It was proven that after 24 h of modification, the samples with these nanoparticles had unnoticeable rubbing. Resistance to bending performed on the samples showed a positive effect of surface modification on the uncoated paperboard with the addition of all observed nanoparticles and a positive effect of the addition of anatase TiO_2_ in the inks applied on coated substrate.

The results of color measurement showed that the addition of rutile TiO_2_ and ZnO nanomaterials in the base ink and surface modification have a positive effect on the color properties of the printed ink films and can be used to improve the UV protection of the observed ink films.

The measurements performed showed that the surface modification improved the adhesion between the “ink–substrate” pairs and had a significant effect on the mechanical and optical stability of the observed ink films. These results may be useful to enable functional print on packaging paperboard substrates. On the other hand, they may be useful when printing different types of inks as a primer for other coatings or varnishes underneath to achieve optimal adhesion and stability between the printed ink film layers.

## Figures and Tables

**Table 1:** Basic properties of TiO_2_ and ZnO nanoparticles.

Production Name		CAS No.	Average Primary Particle Size [nm]	Weight [%]
TiO_2_	Titanium (IV) oxide, anatase	1317-70-0	15	99.7
TiO_2_	Titanium (IV) oxide, rutile	13463-67-7	<100	99.5
ZnO	Zinc oxide	1314-13-2	40–100	>95

**Table 2:** Caliper and grammage of paperboards.

Paperboards	Caliper (mm)	SD	Grammage (g/m^2^) ISO 536	SD
Uncoated	0.4418	0.0029	113.797	0.0476
Coated	0.3595	0.0038	147.35	0.0295

**Table 3:** Adhesion parameters on uncoated paperboard.

Uncoated Paperboard	*γ*_12_ [mJ/m^2^]	*W*_12_ [mJ/m^2^]	*S*_12_ [mJ/m^2^]
Ink + 0%	5.800	62.380	9.280
0.5% TiO_2_ (A)	3.710	66.220	13.120
1% TiO_2_ (A)	2.873	66.807	13.707
1.5% TiO_2_ (A)	3.104	65.286	12.186
0.5% TiO_2_ (R)	6.971	63.059	9.959
1% TiO_2_ (R)	9.167	63.433	10.333
1.5% TiO_2_ (R)	11.234	63.016	9.916
0.5% ZnO	4.755	63.705	10.605
1% ZnO	3.038	66.282	13.182
1.5% ZnO	3.937	67.123	14.023

**Table 4:** Adhesion parameters on coated paperboard.

Coated Paperboard	*γ*_12_ [mJ/m^2^]	*W*_12_ [mJ/m^2^]	*S*_12_ [mJ/m^2^]
Ink + 0%	6.233	62.587	14.187
0.5% TiO_2_ (A)	6.148	62.682	14.282
1% TiO_2_ (A)	6.373	62.637	14.237
1.5% TiO_2_ (A)	3.104	65.286	12.186
0.5% TiO_2_ (R)	5.875	62.955	14.555
1% TiO_2_ (R)	5.994	62.516	14.116
1.5% TiO_2_ (R)	5.760	62.920	14.520
0.5% ZnO	6.719	63.061	14.661
1% ZnO	6.729	62.971	14.571
1.5% ZnO	6.873	62.997	14.597

**Table 5:** Results of the visual assessment of rub resistance.

Deposited Ink Films	Uncoated Paperboard	Coated Paperboard
Ink + 0%	1	1
0.5% TiO_2_ (A)	4	2
1% TiO_2_ (A)	1	1
1.5% TiO_2_ (A)	4	4
0.5% TiO_2_ (R)	4	3
1% TiO_2_ (R)	4	4
1.5% TiO_2_ (R)	4	2
0.5% ZnO	4	4
1% ZnO	4	4
1.5% ZnO	1	2

**Table 6:** Results of the CIE L*a*b* values and color density.

Deposited Ink Films	Uncoated Paperboard				Coated Paperboard			
	L*	a*	b*	*D*	L*	a*	b*	*D*
Ink + 0%	24.929	1.216	–0.284	1.836	5.533	–0.222	–0.966	2.919
0.5% TiO_2_ (A)	24.663	1.053	–0.662	1.825	6.408	–0.083	–1.086	2.916
1% TiO_2_ (A)	25.67	1.344	–0.236	1.827	6.031	–0.141	–1.156	2.901
1.5% TiO_2_ (A)	25.175	1.071	–0.638	1.769	6.5	–0.119	–1.165	2.872
0.5% TiO_2_ (R)	25.715	1.084	–0.324	1.734	5.44	–0.25	–0.962	2.926
1% TiO_2_ (R)	25.139	1.119	–0.499	1.785	5.448	–0.215	–1.054	2.913
1.5% TiO_2_ (R)	24.928	1.072	–0.504	1.801	5.693	–0.246	–1.059	2.9
0.5% ZnO	25.887	1.275	–0.272	1.808	5.72	–0.202	–1.046	2.942
1% ZnO	24.458	1.008	–0.622	1.823	5.537	–0.21	–1.158	2.972
1.5% ZnO	25.92	1.222	–0.236	1.783	5.75	–0.158	–1.224	2.925

**Table 7:** Results of the SFE of ink films.

Uncoated Paperboard	Surface Modification (h)	*γ* ^total^	*γ* ^D^	*γ* ^P^	Coated Paperboard	Surface Modification (h)	*γ* ^total^	*γ* ^D^	*γ* ^P^
Ink + 0%	0	41.63	34.7	6.93	Ink + 0%	0	44.62	40.16	4.46
	12	37.91	37.57	0.34		12	36.31	31.63	4.68
	24	36.27	36.09	0.18		24	35.77	30.09	5.67
1% TiO_2_ (A)	0	43.13	40.95	2.18	1% TiO_2_ (A)	0	44.81	40.44	4.37
	12	38.21	36.01	2.20		12	41.31	35.37	5.94
	24	36.86	36.59	0.27		24	40.00	30.13	9.87
1% TiO_2_ (R)	0	46.05	35.41	10.64	1% TiO_2_ (R)	0	44.31	39.7	4.62
	12	40.14	39.24	0.90		12	40.36	35.49	4.87
	24	39.72	39.11	0.61		24	37.96	31.67	6.29
1% ZnO	0	42.77	40.19	2.57	1% ZnO	0	45.5	41.27	4.24
	12	39.2	38.37	0.83		12	38.69	31.99	6.70
	24	39.75	39.27	0.48		24	39.41	29.79	9.61

**Table 8:** Results of the visual assessment of rub resistance on unmodified and modified samples.

Ink Film	Uncoated Paperboard			Coated Paperboard		
	Surface Modification (h)					
	0	12	24	0	12	24
Ink + 0%	1	2	3	1	1	1
1% TiO_2_ (A)	1	3	3	1	1	1
1% TiO_2_ (R)	3	4	1	5	1	1
1% ZnO	4	4	1	4	1	1

## Data Availability

The authors confirm that the data supporting the findings of this study are available within the article. The raw data that support the findings of this study are available from the corresponding author, S.M.P., upon reasonable request.
